# Does Recall after Sleep-Dependent Memory Consolidation Reinstate Sensitivity to Retroactive Interference?

**DOI:** 10.1371/journal.pone.0068727

**Published:** 2013-07-09

**Authors:** Gaétane Deliens, Rémy Schmitz, Isaline Caudron, Alison Mary, Rachel Leproult, Philippe Peigneux

**Affiliations:** UR2NF - Neuropsychology and Functional Neuroimaging Research Group at CRCN - Center for Research in Cognition and Neurosciences, Université Libre de Bruxelles (ULB) and UNI - ULB Neurosciences Institute; Brussels, Belgium; Hôpital du Sacré-Coeur de Montréal, Canada

## Abstract

Previous studies have shown that newly encoded memories are more resistant to retroactive interference when participants are allowed to sleep after learning the original material, suggesting a sleep-related strengthening of memories. In the present study, we investigated delayed, long-term effects of sleep vs. sleep deprivation (SD) on the first post-training night on memory consolidation and resistance to interference. On day 1, participants learned a list of unrelated word pairs (AB), either in the morning or in the evening, then spent the post-training night in a sleep or sleep deprivation condition, in a within-subject paradigm. On day 4, at the same time of day, they learned a novel list of word pairs (AC) in which 50% of the word pairs stemmed with the same word than in the AB list, resulting in retroactive interference. Participants had then to recall items from the AB list upon presentation of the “A” stem. Recall was marginally improved in the evening, as compared to the morning learning group. Most importantly, retroactive interference effects were found in the sleep evening group only, contrary to the hypothesis that sleep exerts a protective role against intrusion by novel but similar learning. We tentatively suggest that these results can be explained in the framework of the memory reconsolidation theory, stating that exposure to similar information sets back consolidated items in a labile form again sensitive to retroactive interference. In this context, sleep might not protect against interference but would promote an update of existing episodic memories while preventing saturation of the memory network due to the accumulation of dual traces.

## Introduction

Accumulating evidence suggests that sleep plays a promoting role in the off-line consolidation of recently acquired memories [1]. This consolidation process progressively converts labile memory traces into more stable representations in long-term memory, purportedly becoming more resistant to ongoing retroactive interference by similar material [2]. Retroactive interference [3] refers to the phenomenon by which storage of new experiences interferes with previously encoded similar memories, as illustrated by the observation that recalling a memorized item is more difficult when the retrieval cue has been associated with another memory item during the retention interval. That sleep protects memories against interference eventually leading to diminished forgetting over time was already proposed in 1924 by Jenkins and Dallenbach [4], who observed better retention of a learned list of unrelated words after a period of sleep than after an equivalent period spent awake. In their view however, sleep played no more than a passive role in protecting the retention processes from harmful interferences arising from exposition to new information during wakefulness, thus providing a temporary respite for the newly formed memory traces. Notwithstanding, the view of a genuinely passive role of sleep for protecting novel memories and for consolidating memory has been challenged by subsequent studies. Indeed, it has been shown that memory retention is better when sleep takes place just after learning than just before to recall during a similar 24-hours retention interval [5], [6]. Retention of word pairs in verbal long-term memory is also improved after sleep, as compared to wakefulness only when participants sleep in the first part of the night, richer in slow-wave sleep, but not when they sleep during the second half of the night, richer in rapid eye movement sleep (e.g. [6], [7]). If the temporal location of the sleep episode and the distribution of sleep stages induce differential effects on recall performance, then it entails that more complex, active sleep mechanisms are at play to support the consolidation of novel memory traces, than a mere passive protection against interference.

If sleep effectively promotes memory consolidation and makes memories more robust, it also entails that those memories consolidated during sleep should be less easily disrupted by the ongoing presentation of interfering material on the next day. This hypothesis was tested by Ellenbogen et al. [8] using a classical AB–AC interference paradigm. On the first session, participants had to memorize a list of word pairs (AB), after which they spent a period of time either asleep (at night) or awake (during daytime). On the second session, and before retrieval of list AB, half of the participants had to memorize a series of novel word pairs (AC), in which the first word of the pair was the same than in list AB, hence creating interference, whereas the other half directly proceeded to cued recall of list AB. Results showed a better recall of list AB after sleep than wakefulness for material submitted to interference (i.e. recalling list AB just after learning list AC), and more so for non interfering material. This effect was confirmed in a subsequent study in which participants were tested first in non-interfering then in interfering conditions within the same retrieval session [9]. Hence these studies suggested that sleep consolidates new information but also protects this information against retroactive interference.

In the present study, we extended further this perspective by investigating delayed, long-term effects of sleep deprivation on the first post-training night on memory consolidation and resistance to interference in a within-subject paradigm. Participants were tested twice at two weeks interval, once in a post-training sleep and once in a post-training sleep deprivation conditions. All participants were allowed to sleep two supplementary nights after the first post-training night to ensure comparability between conditions. In addition, only half of list AB was subjected to AC interference to allow within-subject comparison of the interference-related effect and to control for individual learning levels. Finally, half of the participants learned the AB list in the morning and the other half in the evening to test for a possible effect of trace decay during daytime on sleep-dependent memory consolidation, as previously found by Gais et al. [6]. Improved recall performance and reduced interference effect were expected after a post-learning sleep period as compared to the sleep deprivation condition.

## Methods

### Participants

#### Ethics statement

The experiment was conducted in accordance with the Declaration of Helsinki and approved by the institutional ethics committee (Université Libre de Bruxelles). Written informed consent was obtained from all participants previous to the study.

This study included twenty-nine healthy French-speaking, right-handed participants (12 males, 21.86±2.75 years, mean ± SD) with intermediate or neutral chronotype (Morningness-Eveningness Questionnaire [10]; range 31–63) and no medical history of neurological disorders, mood troubles, or sleep disturbances (Pittsburgh Sleep Quality Index [11] (PSQI) total score <7). Subjects were required to keep a regular sleep pattern during the week before and throughout the experiment and to refrain from alcohol and stimulant drinks. To control for the regularity of sleep habits, they were asked to wear an actimetric device (Daqtometer, Daqtix GbR, Oetzen, Germany) on the non-dominant wrist during one week before the beginning of the testing throughout the end of the protocol. Participants were instructed to wear this device all the time except when taking a shower. Average wrist movement activity and light variations were recorded every 30 seconds. Subjects were also asked to complete daily sleep logs (St Mary’s Hospital sleep questionnaire [12]).

### Material

A computerized AB–AC interference paradigm was adapted from Ellenbogen et al. [8]. In this paradigm, participants had to learn an AB list followed by an interference list (AC). As our protocol consisted of a within-subjects design featuring a sleep and a sleep deprived conditions, two versions of each list were created, resulting in 4 different lists (AB1, AC1, AB2, AC2) of 28 semantically unrelated French bisyllabic word pairs, emotionally neutral and matched for lexical frequency [13], imageability and concreteness [14]. Lists AC1 (resp. AC2) included 50% of word pairs for which the initial word was presented in list AB1 (resp. AB2) but was associated with a new word (example: AB list: cheval – salon [horse - lounge]; AC list: cheval – canal [horse - channel]), hence creating interference. The other 50% in list AC1 (resp. AC2) were new word pairs. Consequently, during delayed recall of list AB after learning the corresponding list AC, 50% of the word pairs in the list AB were subjected to retroactive interference from the list AC, whereas the other 50% were not. During learning and recall sessions, word pairs of each list were randomly intermixed. AB2 and AC2 lists were paralleled versions of AB1 and AC1 lists, alternatively used for the sleep condition (SH) and the sleep deprived (SD) condition. Conditions and list versions were counterbalanced across subjects.

### Procedure

**Figure 1:**
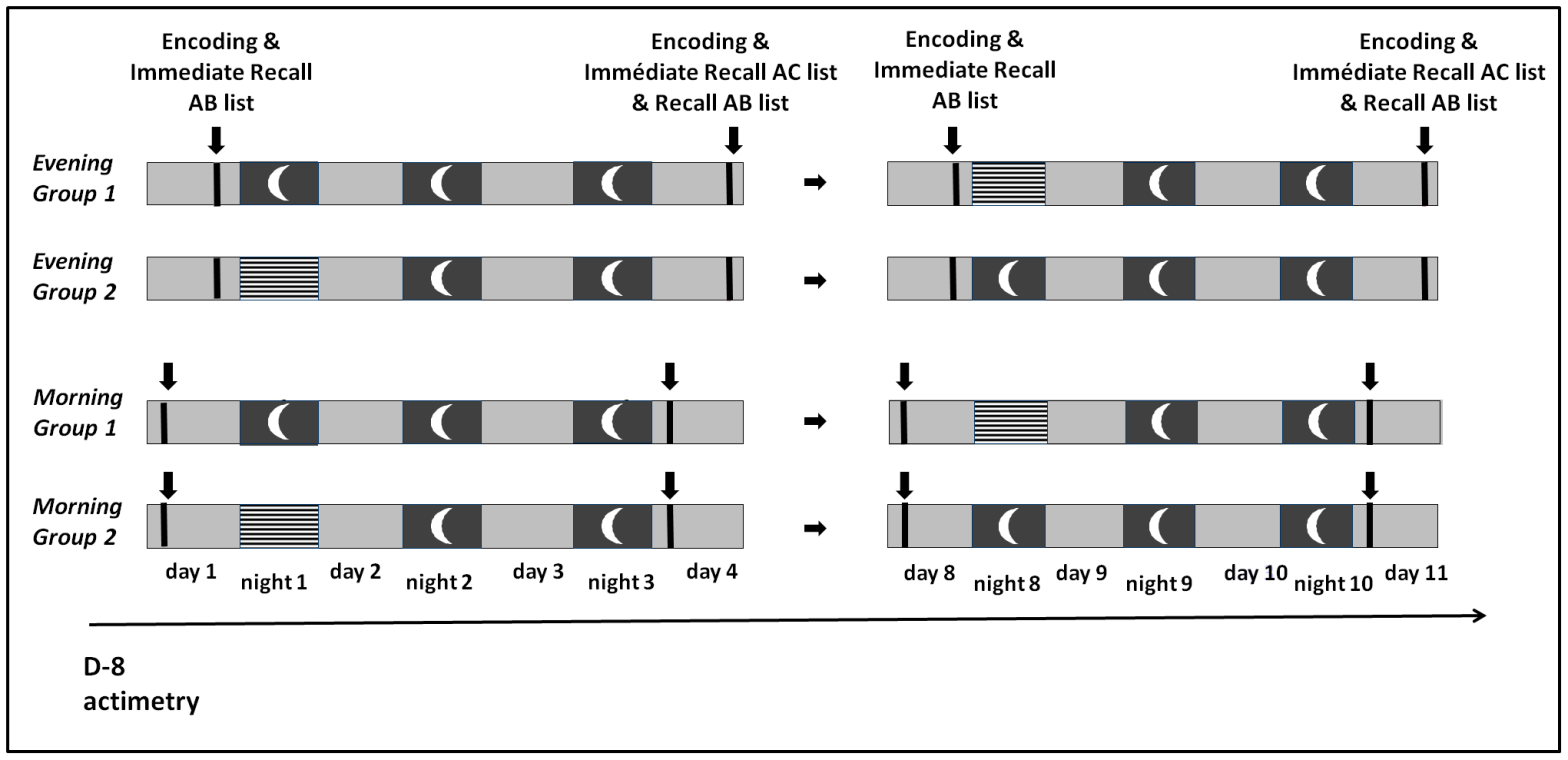
Randomized cross-over design with post-training sleep at home (SH) or sleep deprivation (SD). Group 1 was in the SH condition on the first week of testing and in the SD condition on the second week (conversely for Group 2). Hatched rectangles represent the sleep deprivation nights. (AB) Initial word pairs list; (AC) interference word pairs list. Learning and testing sessions were administered at the same time of day (either learning and retrieving in the morning, or learning and retrieving in the evening) to avoid circadian confounders.

An overview of the experimental design is illustrated Figure 1. At day 1, participants learned either in the morning (M group) or in the evening (E group) the AB list up to the learning criteria (75% of correct responses). Half of them then slept at home (SH) whereas the other half was sleep deprived (SD) during the whole night under the experimenters’ supervision. Participants in the sleep deprivation condition were kept in a room for the whole night. They were allowed to engage in quiet activities (e.g. watching movies, playing society games, discussion group). Water was freely available. No caffeine or stimulant drinks were allowed. A vigilance task was administered every hour. During that night, no participant showed signs of engaging in explicit rehearsal/retrieval practice of the material acquired during the learning session. Afterwards, all participants slept for 2 nights at home, then learned the AC list (again until the 75% learning criteria) on day 4, at the same time as learning list AB (M vs. E). In the ensuing cued recall testing, the first word of the pair was presented and participants had to recall the associated word from list AB only. The following week, they repeated the same procedure using a new list in the other post-training condition (SH or SD).

The 28 unrelated word pairs (of AB and AC lists) were displayed one by one on the computer screen for 3 s, followed for 5 s by a white fixation cross turning red to warn the subject of the transition to the next pair. Immediately after the learning session, an immediate cued recall procedure was administered: the first word of each pair appeared on the computer screen and subjects had to recall the associated word. The correct word pair was then presented on the screen. The cued recall procedure was repeated until the subjects were able to recall at least 21 word pairs (75% learning criterion). When a pair was correctly completed, it was not presented anymore in the next trials to avoid over-consolidation. The sequence of word pairs presentation changed over repeated trials to prevent serial learning.

The cued delayed recall phase was administered immediately after learning the AC list. Cued delay recall was administered using the same procedure than for learning except that pair cues were presented only once. Feedback on correct performance was provided as well.

Additionally, the psychomotor vigilance task [15] (PVT) and the Karolinska Sleepiness Scale [16] (KSS) were administered before each learning or test sessions, as well as throughout the sleep deprivation night to estimate objective and subjective vigilance levels, respectively.

## Results

### Sleep and Vigilance Variables

First, we conducted first a visual inspection on actimetric recordings and controlled for their concordance with self-reported questionnaires. Based on this analysis, 5 participants were excluded due to irregular sleep patterns (<6 hours of sleep during the night before learning and recall sessions, bedtime after 2 am) and one due to technical failure. For the 23 remaining volunteers, actimetric data were analysed for the four days and nights preceding the learning episode in both the SH and SD conditions. Actimetric values recorded every 30 seconds were hourly averaged then further summarized into Day and Night mean activity values, and entered in a repeated measures ANOVA with within-subject factors Night (nighttime [8 hours] vs. daytime [16 hours] period), Condition (SH vs. SD) and Cycle (days 1 to 4 before learning). This analysis revealed a main effect of Night with higher mean activity during daytime than during nighttime (mean activity ± standard deviation 1894±114 vs. 392±24, p<.0001). All other effects and especially interactions with the factor Condition were non-significant (ps >.35). Additionally, we computed in the SH and SD conditions the acrophase (15.3±1.9 vs. 15.3±1.5 hour), mean (123±32 vs. 129±34) and amplitude (381±95 vs. 394±110) of activity variations (summarized over successive 6-minutes epochs) for the four same 24-hours cycles (using Acro 3.5 software [Refinetti, 2004; http://www.circadian.org]). T-tests for dependent samples conducted on these variables in SH vs. SD conditions were all non-significant (all ps >.23). Additionally, we computed a repeated measures ANOVA on averaged hourly activity with within-subject factors Night (nighttime [8 hours] vs. daytime [16 hours] period), Condition (SH vs. SD) and Recovery (days 1 vs. 2 after the SH or SD post-learning manipulation). This analysis only revealed a main effect of Night with higher activity during daytime than during nighttime (p<.0001; all other effects ps >19). Altogether, these results suggest a similar activity cycle before learning and before recall in the SH and SD conditions. Moreover, mean sleep duration within the month preceding the experiment, as indicated by PSQI (duration: 7.90±0.58 hours), did not significantly differ from daily measures collected using the St Mary’s Hospital sleep questionnaire (duration: 8.18±0.49 hours; t(22) = 1.99, p = 0.06) during the whole testing period.

A repeated measures ANOVA on mean self-reported sleep duration during the night preceding the experiment with factors Sleep (SH vs. SD) and Session (Learning vs. Recall) did not reveal any significant effect (Sleep: F(1,22) = 0.99, p = 0.33; Session: F(1,22) = 0.67, p = 0.42; interaction: F(1,22) = 0.004, p = 0.95), indicating similar mean sleep durations before learning and recall sessions in both conditions. During the first night after learning in the sleep condition, mean sleep duration was 7.91±1.12 hours. A repeated measures ANOVA on self-reported sleep duration for the two recovery nights in the sleep and wake conditions was computed. Results disclosed a main effect of the recovery night (F(1,22) = 9.5, p<.01), a main effect of sleep conditions (F(1,22) = 21.6, p<. 001) and an interaction (F(1,22) = 29.4, p<0.001). As expected, the mean duration of the first recovery night was longer in the SD condition (10 hr 42 min ±2 hr) than the first recovery night in the SH condition (7 hr 48 min ±1 hr 18 min) and the second recovery night both in the SH (8 hr 06 min ±1 hr 14 min) and in the SD (8 hr 23 min ±1 hr 13 min) conditions (all ps <0.001). However, the duration of the second recovery night, before retesting, was similar in the SH and SD conditions (p>.8). Hence our data show the expected rebound after sleep deprivation, but also a normalisation of the sleep pattern (in terms of duration) already on the following night.

Mixed measures ANOVAs performed on vigilance scores assessed by KSS with the within-subject factors Sleep (SH vs. SD) and Session (Learning vs. Recall) and the between-subject factor Time of learning (morning [M; *n = *7] vs. evening [E; *n = *16]) did not reveal any significant effect of Sleep, Time of learning, Session or any interaction (all p-values >0.1) indicating that subjective vigilance levels during the learning and recall phases were similar in the SH and SD conditions. Similar analysis conducted on PVT data (mean RTs) revealed a main effect of Time of learning (F(1,21) = 12.71, p = 0.002) with shorter mean reactions times in the morning (274.71±9.39) than in the evening (314.83±6.21) group. All other main effects or interactions were non significant (all ps >.05). ANOVAs conducted on PVT median reactions times also revealed a main effect of Time of learning (F(1,21) = 9.87, p = 0.005) with shorter median reactions times in the morning (274.73±9.00) than in the evening (308.67±5.96) group. All other main effects or interactions were non significant (all ps >.09). ANOVAs conducted on numbers of lapses defined as the RTs above 500 msec or as the number of lapses with RTs higher than 2 standard deviations of the mean also failed to disclosed any main effect or interaction (all p-values >0.1).

### Encoding Data

A repeated measures ANOVA conducted on the number of trials needed to reach the learning criteria (75% of correct answer) at immediate recall with the within-subject factors Sleep (SH vs. SD) and Session (List AB vs. List AC) and the between-subject factor Time of learning failed to disclose any main or interaction effects (Morning group SH condition: List AB 2±0.8, List AC 2±0.6; SD: List AB 2.1±0.9, List AC 2.1±1.1; Evening group SH condition: List AB 1.7±0.9, List AC 1.9±0.6; SD: List AB 1.8±0.8, List AC 1.9±0.9; all p-values >.4).

### Interference Paradigm

Across all conditions, the mean number of correctly recalled word pairs was 9.57±6.87 (i.e. 34.18% ±24.54% in percent of maximum recall) in the SH condition and 10.74±7.12 (38.36% ±25.43%) in the SD condition. Pearson’s correlation between recall performance in the SH and SD condition was highly significant (r = 0.61, p = 0.002), suggesting that individual learning abilities similarly influenced recall levels in the two experimental conditions.

**Figure 2:**
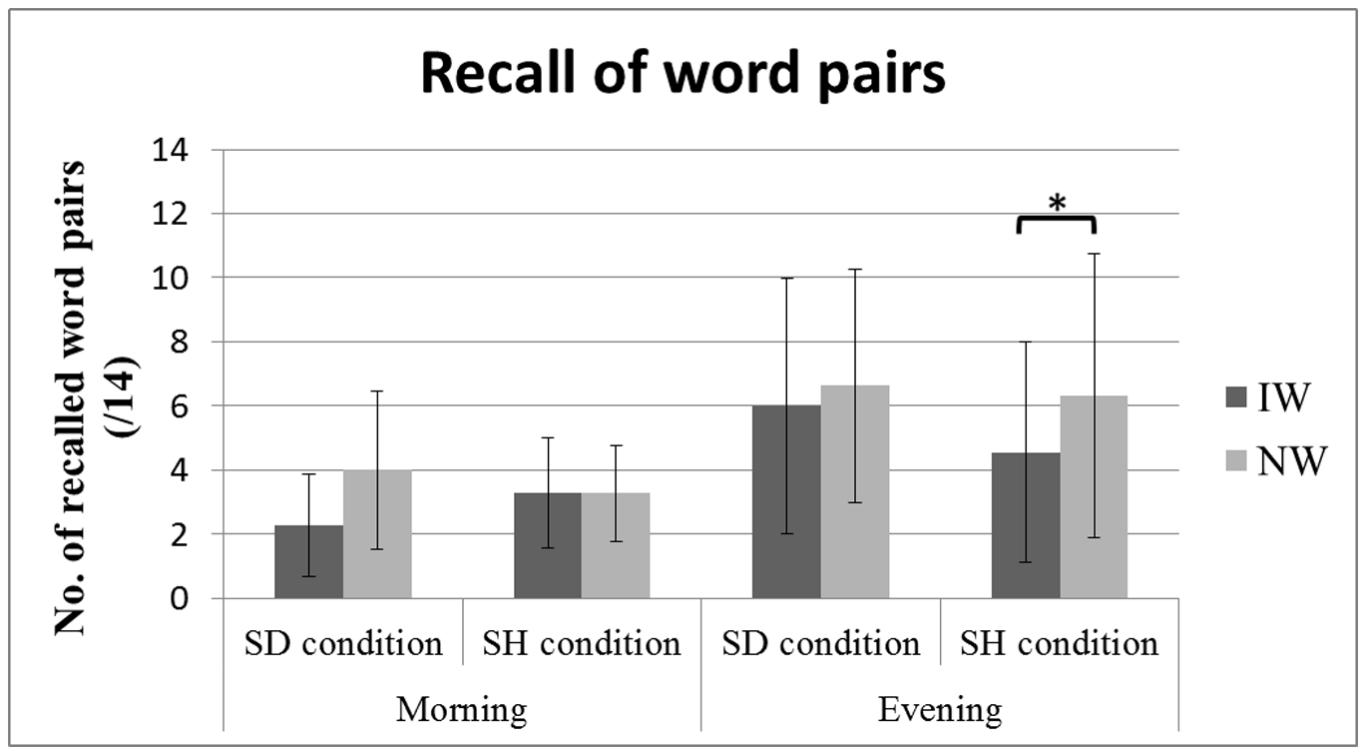
Recall scores (number of correctly recalled words from list AB) for pairs subjected (IW) and not subjected (NW) to interference in SD and SH conditions, in morning and evening groups. Error bars indicate standard deviations.

A mixed measures ANOVA on the number of correctly recalled word pairs with the within-subject factors Sleep (SH vs. SD) and Interference (word pairs from the AB list subjected [IW] vs. not subjected [NW] to interference), and between-subject factor Time of learning (Mvs.E) (Figure 2) disclosed a main Interference (F(1,21) = 18.75, p<0.001) and a triple Sleep*Interference*Time of learning interaction (F(1,21) = 4.65, p = 0.043) effects, but no main effect of Sleep (F(1,21) = 0.26, p = 0.61). The main effect of Time of learning was nearly significant (F(1,21) = 3.99, p = 0.059) with a better recall performance in the evening-learning group (11.78±7.54; 42.07%±26.93%) than in the morning-learning group (6.43±3.18; 22.96%±11.36%). Bonferroni-Holm multistage post-hoc tests revealed a significant interference effect after controlling for multiple comparisons in the evening-learning [E] group, with a lower correct recall for IW (4.56±3.44; 32.57%±24.57%) than for NW (6.31±4.42; 45.07%±31.57%) pairs. No significant difference was present after multiple comparisons neither in the [E] SD condition (IW 6.00±3.98, 42.86%±28.43% vs. NW 6.62±3.65, 47.29%±26.07%, p = 0.92), nor in any condition in the morning-learning [M] group (SH: IW 3.29±1.70, 23.5%±12.14% vs. NW 3.28±1.50, 23.43%±10.71%; SD: IW 2.29±1.6, 16.36%±11.43% vs. NW 4±2.45, 28.57%±17.5%).

## Discussion

The present study investigated stimulus-related retroactive interference effects on the delayed recall of memories consolidated during sleep or wakefulness. In line with a prior report [6], we found a trend for a better delayed recall of learned word pairs when learning occurred in the evening, closer to the usual bedtime, than when it occurred in the morning. This result is in agreement with the seminal study of Jenkins and Dallenbach (1924) [4], in that novel information is progressively forgotten with time elapsed during time spent awake according to the Ebbinghaus' forgetting curve [17], but that the occurrence of sleep stabilizes memories at the pre-sleep level. In the sleep-deprived condition however, performance was higher in the evening-learning than in the morning-learning group, but the duration of the retention interval was similar. Therefore, differences in recall performance between the morning and evening sleep-deprived conditions cannot be solely attributed to a mere time-dependent degradation of the memory trace during wakefulness. An additional contribution for this effect could include a larger amount of diversion interference [18] that took place between the encoding session and the first sleep episode in the morning group. The concept of diversion interference, derived from Muller and Pilzecker [3], and developed by Dewar et al. [18], proposes that any post-learning mentally effortful interpolated task, irrespective of its content, can induce forgetting and retroactive interference effects. In this respect, it is likely that participants in the morning-learning condition have been exposed to higher levels of diversion interference over the period that immediately followed learning during daytime than participants in the evening-learning condition who have been kept awake in quiet controlled conditions during the whole night. In addition, a lack of a sleep-dependent effect for the remaining memories at the time of sleep in the morning-learning group can probably be ascribed to a floor effect as the recall performance was very low in both SH and SD conditions (2 to 4 recalled word pairs out of 28). A more stringent learning procedure in the morning-learning group (95% learning criterion) may have allowed an effect of post-training sleep on the consolidation of residual memory traces, a hypothesis that should be tested in further studies.

Most importantly, we found an effect of retroactive interference in evening-learning participants when they were allowed to sleep after learning, but not when they were sleep-deprived, contrary to our initial expectations and to the results of the Ellenbogen et al. study [9] suggesting that sleep protects against interference. Methodological differences between the two studies may partly explain these discrepancies. First, Ellenbogen et al. [9] used a between-subject design to compare the sleep and the wake conditions whereas our subjects underwent both conditions, accounting for inter-individual differences in declarative learning abilities. Indeed, it is well known that individuals differ in their capacity to learn and memorize verbal material, even within the boundaries of normality. In this respect, the purpose of our within-subjects design was to control for inter-individual differences in learning abilities, by having the same subjects exposed to the two experimental conditions. Furthermore in the present study, the total number of word pairs recalled in the sleep condition was positively correlated with the total number of word pairs recalled in the sleep deprived condition, suggesting a good within-participants stability in learning levels across conditions. Second and more importantly, our subjects were tested on day 4 after a post-training night of sleep deprivation followed by two recovery nights (3 nights of sleep in the sleep condition), whereas participants were tested after a 12-hour interval spent awake during daytime or including a sleep period during the night in the Ellenbogen’s study [9]. Gais et al. [6] already showed that testing memory retention directly after sleep deprivation when participants are under acute fatigue might impact on the results. In this context, reduced memory performance observed in the wake condition of Ellenbogen's study [9] may partly reflect deficits in retrieval due to fatigue and sleepiness rather than a genuine impairment of memory consolidation [7], [19]. The two recovery nights before testing memory retention allowed us to rule out fatigue accumulation during sleep-deprivation as confounders. Our results also feature delayed effects of post-learning sleep deprivation on memory performance, as compared to a more proximate retrieval observed after 12 hours spent awake or asleep. Finally, although Ellenbogen et al. [9] did not evidence circadian effects on learning (the learning performance was similar in the wake/day and the sleep/night groups), an effect of the circadian process on recall abilities cannot be entirely excluded. Our experimental design, including total sleep deprivation and delayed testing, was designed to control for these potential circadian effects. Further studies should investigate these potentially confounding factors.

In the evening-learning group, our results showed a retroactive interference effect after post-training sleep but not after wakefulness. Although these results might suggest that sleep does not enhance the strength of the memory against retroactive interference, we propose that these findings are best explained within the framework of the memory reconsolidation theory [20], [21], hypothesizing that the recall of previously stored information puts them back in a labile form, again sensitive to interference. In this perspective, the reactivation of sleep-consolidated AB associations following presentation of the “A” word while learning novel but related AC material would set back AB memory traces in a labile form that can be disrupted by the AC association. This would also explain, in our results, why we found similar recall of word pairs subjected to interference in the sleep and the sleep deprivation conditions. Indeed, presentation of “A” words in the AC list would automatically reactivate the corresponding B words for sleep-consolidated memories. These AB associations would then be disrupted by learning the AC pairs, thus eventually dampening the recall performance to the level of recall after post-training sleep deprivation.

The mechanisms by which sleep makes memory traces sensitive to reactivation and reconsolidation remain a matter of debate. Consistent evidence supports the hypothesis that memory consolidation processes are promoted during sleep (for a review see [1]), and that the reconsolidation theory implicitly assumes that to-be-reactivated (and possibly destabilized) memories must have been previously consolidated. In this context, it can be argued that sleep may selectively set memory traces in a state sensitive to updating through the reconsolidation process [22]. Following this interpretation, rapid cellular consolidation processes would strengthen memory traces immediately after learning [23]. Slower subsequent processes, e.g. processes subtending the strengthening of neocortico-cortical connections concomitantly with the weakening of hippocampo-cortical connections [24], preferentially occurring during sleep, would then allow more substantial changes to be written at the system's levels. Being consolidated, the trace (i.e. the learned AB pair) would be susceptible to reactivation and destabilisation upon partial presentation (e.g. the word A) when exposed to the interfering AC pair. In this respect, sleep would promote the consolidation of memories and the transformation of these memories into a format that remains sensitive to reactivation/destabilization, hence still updatable.

Mounting animal and human evidence supports the reconsolidation theory (see for e.g. [21], [22], [25]). For instance in rats, injection of a protein-synthesis inhibitor (i.e. blocking consolidation) disrupts an already established long-term fear conditioning, but only if the reminding conditioned stimulus is presented before the injection [25], [26]. This indicates that reactivation of the fear conditioning sets the memory back to an unstable form, requiring protein-synthesis to be “reconsolidated” again, a process prevented by the injection of a protein-synthesis inhibitor. In humans, after learning a pre-defined sequence in a procedural memory finger-tapping task on day 1, a brief rehearsal of the same sequence on the next day (day 2) just before learning a novel sequence actually impairs delayed recall performance for the first sequence on day 3, as compared to a group who did not rehearsed the initial sequence before learning the novel one [27]. At variance, alterations in performance were not observed when retest of the first sequence occurred just after learning the second sequence. This suggests that the presentation of the second sequence following the reactivation of the first one exerts a blocking role on subsequent reconsolidation, rather than immediately reversing the learning of the first sequence. Likewise in the episodic memory domain, after learning a set of objects on day 1, repetition of the encoding procedure for the same set of objects immediately before learning a novel set of objects on day 2 impairs recall performance on day 3 [28]. The statement that interfering learning-related harmful effects on recently reactivated memories need time between the learning of AC list and the recall of AB list has not been directly validated. In a recent report however ([29]; quoted in [22]), participants learned a list of items (list 1), then slept or were sleep deprived during the post-learning night. After recovery sleep, they were reminded of list 1 before learning a novel list of items (list 2). Immediately after learning list 2, they had to recall list 1. In line with our results, this study found an interference effect after a night of normal sleep, but not after a night of sleep deprivation. At first glance, these findings may be interpreted as immediate effects of an interfering learning on recently reactivated memories. Based on these results however, Nadel et al. [22] surmised that the absence of an interference effect on recently reactivated memories in the sleep-deprived group might be due to a state-dependent memory phenomenon. In the Newman-Smith et al. experiment [29] indeed, subjects learnt list 1 in a well-rested state, but recalled list 1 in a sleep-deprived state, possibly leading to a state-related interference effect. Accordingly, several studies have found diminished recall performance when subjects are in a different state (mood and physical context) at the encoding and retrieval sessions (e.g. [30], [31], [32], [33]. In our study however, the recall session took place after two recovery nights. Therefore, subjects were in a well-rested state at encoding as well as at retrieval, ruling out this state-dependent retrieval interpretation. As polysomnographic recordings were not obtained in the present study, we cannot relate the changes in performance to post-learning sleep variables. This point should be addressed in further studies.

Although the reconsolidation theory provides a good explanation for our findings of an interference effect in the post-learning sleep condition, alternative explanations are possible. In our study, the AC lists contained half of the A-words associated with new C-words; the other half was made of completely new words pairs. Although only half of the AC list contained initial A-words, presentation of interfering A-words might have reactivated the whole AB list under non-consolidated condition, rather than only the associated words. Therefore, after sleep deprivation, both the AB pairs subjected to AC interference and those not directly subjected to interference may have been set again in a labile form because sleep-dependent memory consolidation processes did not take place during the post-learning night. Conversely, after a night of post-learning sleep and of consolidation in long-term memory of the AB list, only the words being part of the initial learning episode and specifically presented during learning of the AC list may have been reactivated, without generalisation to non-presented AB pairs. Following this point of view, post-learning sleep would consolidate memories but also limit the impact of retroactive interference on items directly submitted to interference, by actually preventing “labilisation” of the whole learning episode as it was the case in the sleep deprivation condition.

To differentiate these two competing hypotheses (specific- versus generalized-reactivation of the learning episode), a control group could be added in the sleep and sleep deprived conditions, who will not learn the AC list before the recall of AB list, i.e. a non-interference condition. If learning the AC pairs actually impaired (“labilised”) the whole AB list (including pairs non directly submitted to interference), then recall performance for non interferent word pairs in the AC list learning interference group should be lower than recall performance in the control group, in which no generalization of interference was possible. Conversely in the SH condition in which sleep-dependent consolidation processes should have taken place, if presentation of the AC list leads to the reactivation of only the words pairs associated with A-cues, then recall performance for word pairs non directly submitted to interference should be similar in the group submitted to interference and in the no-interference control group. This hypothesis should be tested in a further study using a similar within-subject approach to control for potential interindividual differences in learning and memory abilities.

To sum up, our results evidence a specific role of post-training sleep for the consolidation of recently learned memories, that we interpret as paradoxically expressed in the reconsolidation phenomenon. Indeed, we have shown a retroactive interference effect suggesting that AB pairs subjected to AC interference are set again in a labile form only in subjects allowed to sleep during the post-training night. By contrast in the sleep deprivation condition, the interference effect was non-significant, although performance was above floor level. We tentatively hypothesize here that since the learned information was less consolidated due to the absence of post-training sleep, a dual memory trace might have been created while learning the AC list, comprising both the AB and AC elements. This double association would thus partially prevent the negative impact of the retroactive interference. Indeed, the second AC list would not markedly modify the first AB list, but the two lists would coexist in memory. Further studies are needed to test this tentative hypothesis as well as the long-term consequences, to the scale of weeks or months, or in terms of underlying functional neural networks, of the inscription of a dual trace in long-term memory after sleep deprivation.
